# P-1482. Interim Safety Results of the Recombinant Zoster Vaccine in Young Adult Solid Organ Transplant Recipients

**DOI:** 10.1093/ofid/ofaf695.1668

**Published:** 2026-01-11

**Authors:** Molly C Schnieders, Rachel Osoba, Emily Shteynberg, Ravi Jhaveri, Taylor Heald-Sargent

**Affiliations:** Ann & Robert H. Lurie Children's Hospital of Chicago, Chicago, IL; Ann & Robert H. Lurie Children's Hospital, Chicago, Illinois; Northwestern University, Chicago, Illinois; Ann & Robert H. Lurie Children's Hospital of Chicago, Chicago, IL; Northwestern University Feinberg School of Medicine, Ann & Robert H. Lurie Children's Hospital, Stanley Manne Children's Research Institute, Chicago, Illinois

## Abstract

**Background:**

Reactivation of Varicella-Zoster Virus (VZV) causes symptomatic illness that can be extreme in patients with an immunocompromising condition like solid organ transplant recipients. ACIP recommendations have endorsed the Recombinant Zoster vaccine (RZV) for immunocompromised patients who are 19 years and older, but detailed immunologic data is still lacking. We are reporting interim safety results from a safety and immunogenicity study of RZV in young adults 19-40 y.o. who are solid organ transplant recipients.

**Figure:**
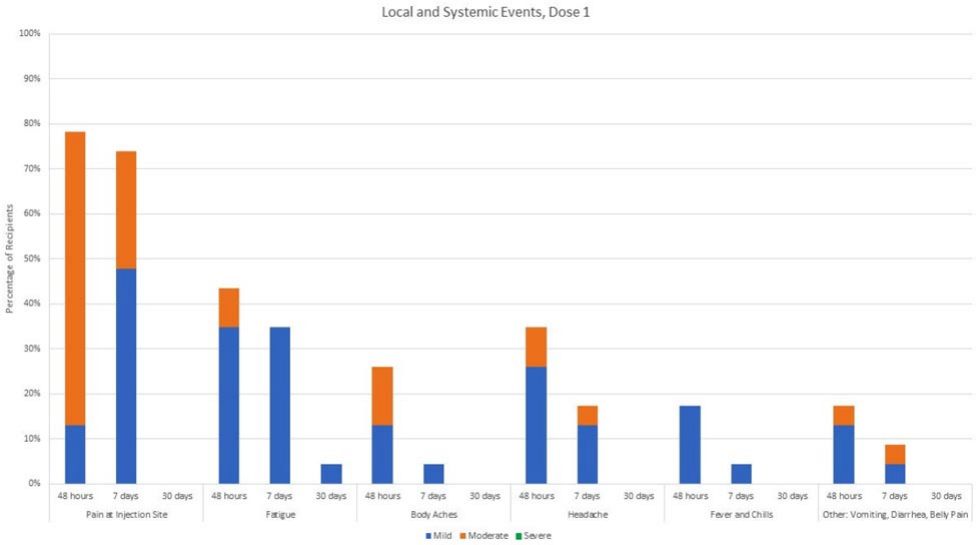

**Figure:**
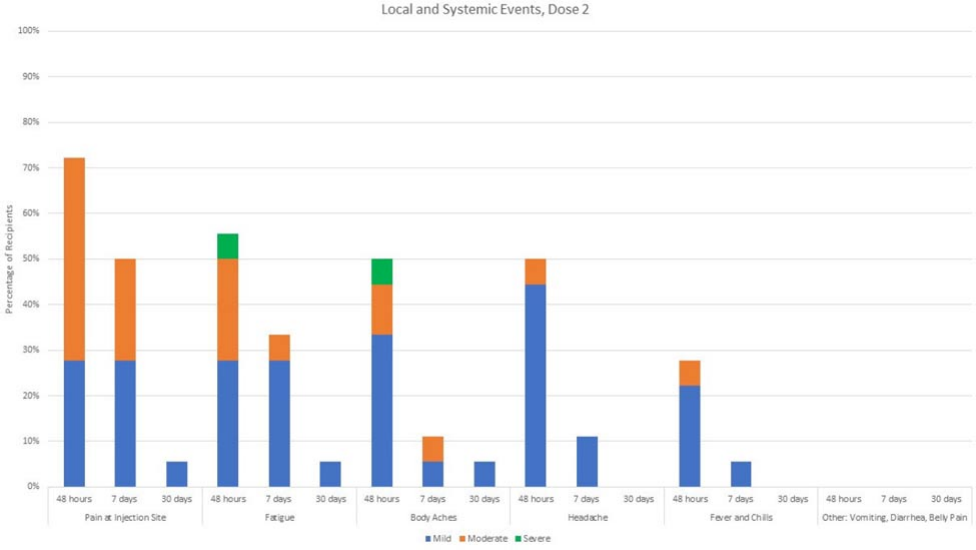

**Methods:**

We screened and consented eligible young adult recipients of solid organ transplants (heart, liver, kidney). Participants receive two doses of RZV, 75 days apart, and complete symptom surveys daily for 7 days then weekly for the remainder of the month. Multiple follow-ups are conducted to assess for any vaccine-related side effects, clinical signs of VZV reactivation, or signs of acute rejection. Symptom data is reported as a percentage of all recipients for dose 1 and dose 2 at 48 hrs, 7 days and 30 days after each dose. Severe adverse events are reported regardless of when they occurred.

**Results:**

Twenty-three participants have received the first dose and 18 participants have received a second dose. The age range was 19-23 y.o. with 14 heart, 6 liver, 4 kidney. Symptoms were generally mild, and the vaccine was well-tolerated. The symptoms are summarized in Figure 1.

One recipient had a severe adverse event with acute onset of fever, chest pain, and shortness of breath within 12 hours of the second dose. The participant visited the ED, improved with fluid resuscitation and was observed before ultimately being discharged. ED testing showed elevated proBNP (2023 pg/ml) but normal Troponin-T. Partial echocardiogram showed mildly decreased right ventricular function and normal left ventricular function. In outpatient clinic 2 days later, their pro-BNP had declined to 684 and their echo still showed mildly decreased RV function. Clinically, aside from some residual fatigue, the patient felt fine. Donor-derived DNA testing at that visit was negative.

**Conclusion:**

RZV has been generally well tolerated in recipients thus far. The one adverse event does not appear to have a lasting impact and should not be a reason to avoid RZV vaccination.

**Disclosures:**

Ravi Jhaveri, MD, AstraZeneca: Advisor/Consultant|Gilead: Advisor/Consultant|GSK: Grant/Research Support|PIDS: editorial stipend-JPIDS|Sanofi: Advisor/Consultant|Seqirus: Advisor/Consultant|UptoDate: royalties Taylor Heald-Sargent, MD PhD, GSK: Grant/Research Support

